# Seed-Assisted Crystallization in the Hydrothermal Synthesis of FAU Zeolite from Acid-Treated Residue Glass Powder

**DOI:** 10.3390/ma18071393

**Published:** 2025-03-21

**Authors:** Paulla B. F. Sousa, Lindiane Bieseki, Sibele B. C. Pergher

**Affiliations:** 1 Laboratory of Molecular Sieves (LABPEMOL), Institute of Chemistry, Federal University of Rio Grande do Norte, Natal 59078-970, RN, Brazil; p_franca_sousa@outlook.com (P.B.F.S.); lindiane.bieseki@gmail.com (L.B.); 2 Laboratory of Catalysis, Instituto Nacional de Tecnologia (INT), Rio de Janeiro 20081-312, RJ, Brazil

**Keywords:** FAU zeolite, industrial waste, colorless glass powder, seed crystals, acid leaching

## Abstract

A simple and low-cost synthesis assisted by seed crystals has been proposed to convert residual colorless glass powder into a Na-X zeolite. For this purpose, the optimal parameters for acid leaching of glass powder were studied to remove impurities that could interfere with the crystallization process. Then, the hydrothermal syntheses were supported by Na-X seed crystals (0% to 5%, wt.) to induce the growth of zeolite X, evaluating the crystallization time (12 h to 48 h) and the variation of the silicon source (acid-treated and untreated residues). The formation of the faujasite as the main phase, with a higher degree of structural order and microporosity, was observed with the previous treatment in the residue, a higher seed loading, and a shorter crystallization time. On the other hand, a phase competition between faujasite, gismondine, Linde type-A, and sodalite structures was observed in the zeolites synthesized from the untreated residue. In this case, the high seed loading and the longer synthesis time allowed the correct targeting of the faujasite structure with low structural order and micro/mesoporous properties. Furthermore, interzeolite transformations occur in all syntheses, where the framework type synthesized was influenced by the presence of a specific ion as a mineralizing agent.

## 1. Introduction

Zeolites are microporous molecular sieves with uniform pore sizes of less than 2 nm (20 Å), whose open 3-dimensional framework structures are composed of corner-sharing tetrahedrons (TO_4_), where these T-atoms can be silicon (Si) or aluminum (Al), known as aluminosilicates [1]. Faujasite (FAU) framework structures have a three-dimensional micropores system with a large pore diameter (0.74 nm), a highly accessible specific area (1608.34 m^2^ cm^−3^), and an accessible volume of around 27% [2]. Among zeolites belonging to the family of FAU structures, zeolite X has a Si/Al molar ratio between 1.0–1.5 [3]. This synthetic zeolite is applied in the separation by adsorption of N_2_ and O_2_ [4], as well as having potential applications in the adsorption of heavy metals [5] in the treatment of water and industrial effluents [6,7].

According to Khaleque et al. [8], synthetic zeolites, compared to natural zeolites, present certain advantages in their production and the targeted application. By requiring a few hours/days of production and by ease in the control of synthesis parameters, the formation of these structures is conducive to obtaining nanomaterials with high purity, chemical and thermal stability, specific surface area, selectivity, and controllable pore size.

However, depending on the nature of the reagent and the industrial-scale zeolite production process, synthesis methodologies for these materials become costly from an economic point of view. One approach to finding production routes with economic advantages is to determine a cost-controlling feature [9]. Thus, raw materials from industrial waste are an alternative source of precursors in the synthesis of low-cost and environmental characteristics. Several authors have studied the synthesis of zeolites by applying raw materials as a source of silicon, such as crushed stone powder and aluminum ash [10], coal fly ash [7], lithium silicon-aluminous residue extracted from α-spodumene [11], and β-spodumene [12], and waste glass [5,13,14,15,16,17,18,19,20,21,22,23,24,25,26,27,28,29,30,31].

Although a structure favorable for zeolite synthesis is chosen based on the chemical composition of the residue studied, it is difficult to produce pure-phase zeolites from waste materials for specific applications, so a pretreatment of the waste material is required [8]. Acid leaching is one of the techniques used to reuse and purify waste materials in producing porous materials composed of silicon and aluminum [32,33].

Furthermore, the use of seeds from the same studied structure is an alternative to properly directing crystal growth to develop more sustainable and economical synthetic routes [9]. This approach has been successfully utilized to synthesize various zeolite frameworks [34,35,36,37]. Seeded crystals can provide interfaces for heterogeneous nucleation by passing the induction period, consequently being able to reduce total synthesis time, eliminate impurities, and alter particle size [38].

In this paper, seed-assisted crystallization was studied in the conversion of colorless glass powder into a large pore zeolite with FAU-X structure by hydrothermal synthesis. The main experimental conditions evaluated were the variation of the silicon source, the amount of seed, and the crystallization time. The molar composition of the synthesis gel and crystallization temperature were kept constant according to the standard method for obtaining zeolite X. In addition, the acid leaching technique was applied to the glass powder to eliminate impurities in the waste material.

Therefore, this work provides insights into the effects of mineralizing agents, crystallization times, and types of silicon sources on directing to a maximum production of a unique-phase value-added Na-X zeolite. Also, this investigation could help direct future research toward the large-scale production of zeolitic materials from industrial wastes.

## 2. Materials and Methods

The materials employed in the experimental procedures were silica (Aerosil 200, Evonik, Essen, Germany), sodium hydroxide (NaOH, Dinâmica Química Contemporânea Ltda, Indaiatuba, SP, Brazil), sodium aluminate (NaAlO_2_, 45% of Na_2_O and 55% of Al_2_O_3_, anhydrous, Riedel-de Haën, Buchs, Switzerland) for synthesis; and hydrochloric acid (HCl, P.A. 37%, Neon, Suzano, SP, Brazil) for the leaching process. The chemicals applied in this work were analytical grade and without further purification.

The residual glass powder (RGP) from cutting and lapping colorless glass was provided by a company in Pernambuco (Brazil). The starting material for the zeolite syntheses was dried at 150 °C and sieved to obtain a grain size of around 300 μm. The chemical composition (%, wt.) of RGP (determined by XRF analysis) were as follows: 66.94% SiO_2_, 3.31% Al_2_O_3_, 8.80% Na_2_O, 14.84% CaO, 3.60% MgO, 0.67% Fe_2_O_3_, 0.67% K_2_O, 1.17% others.

### 2.1. Acid Leaching in Residue Glass Powder

The acid leaching technique’s experimental conditions were evaluated to obtain a silicon source with a lower impurity content.

#### 2.1.1. Study of the Optimal Temperature for Acid Leaching

2.000 g of RGP were leached with 40 mL of HCl solution (2 mol L^−1^), stirring with a reflux system for 265 min at room temperature (25 °C) and at 80 ± 3 °C, nominated as TRGP01_25C and TRGP01_80C, respectively. Thereafter, the treated samples (pH = 0~1) were washed with distilled water, filtered until pH = 5, and dried at 60 °C for 12 h.

#### 2.1.2. Study of the Optimal Experimental Condition for Acid Leaching

2.000 g of RGP were leached in an acid medium with a reflux system at 80 ± 3 °C. The experimental variables studied were HCl solution concentration (0.5 and 3.0 mol L^−1^), liquid/solid ratio of acid solution (20 and 60 mL) to each 2.000 g of RGP, and treatment time (60 and 240 min). At the end of the procedure, the treated samples (TRGP02) were washed with distilled water, filtered until pH = 5~7, and dried at 60 °C for 12 h.

### 2.2. Syntheses of FAU Zeolites

The synthesis methodologies described in this work were performed based on a standardized synthesis of zeolite X-type faujasite (FAU) obtained from the International Zeolite Association (IZA), with adaptations [39].

#### 2.2.1. Synthesis of FAU-X Zeolite Seeds

A portion of NaAlO_2_ was added to the NaOH solution with a molar composition of 0.02 NaAlO_2_:0.09 NaOH:1.97 H_2_O, stirred until dissolution, and reserved. Another homogeneous solution was formed by adding amorphous silica to the NaOH solution (0.07 SiO_2_:0.17 NaOH:4.36 H_2_O) and stirring for 10 min. Afterward, the sodium aluminate solution was slowly poured into the silicon solution and stirred for 30 min at room temperature (25 °C). The formed gel (pH = 14) was transferred to a Teflon-sealed autoclave and carried out to a static heater block for crystallization at 100 °C for 24 h. At the end of the crystallization step, the FAU-X seeds (named ZX) were washed with distilled water until neutral pH, filtered, and dried at 60 °C for 12 h.

#### 2.2.2. Study of Optimal Seed Dosage in the Synthesis of FAU Zeolite with Untreated and Acid-Treated Residues

The aforementioned synthesis of the FAU structure methodology was applied to the study of optimal seed dosage and treatment time. Untreated (sample RGP) and acid-treated residue glass powder (sample TRGP02_6 named TRGP) were used as silicon sources. The amounts (mass, g) of NaOH, distilled water, and NaAlO_2_ were adjusted so that each autoclave had 0.846 g of SiO_2_, considering the chemical composition of the residues glass powder (RGP and TRGP) and maintaining the molar composition of FAU-X zeolite synthesis gel.

After the formation of the amorphous gels, the number of seed (ZX sample) equivalent to 0%, 1%, and 5% (wt.) of solids used in this synthesis (residue, NaOH, and NaAlO_2_) was stirred for 5 min. The mixtures were transferred to a needed number of Teflon-sealed autoclaves and carried to a static heater block to crystallize at 100 °C for 12 h, 24 h, 36 h, and 48 h. Then, the products were washed with distilled water until neutral pH and dried at 60 °C overnight. The samples from different residues (*a* = R (RGP) or TR (TRGP)), the number of seeds (*b* = S0, S1, or S5, with 0%, 1%, or 5% wt., respectively), and crystallization times (*t*) were denoted as ZX *a b*_*t* H.

### 2.3. Characterization of the Samples

Powder X-ray diffraction (XRD) patterns of the samples were performed on a Bruker D2-Phaser diffractometer (Atibaia, SP, Brazil), with Lynxeye detector (Atibaia, SP, Brazil), copper radiation (CuKα, λ = 1.54 Å), filtered by nickel, operating at 10 mA and 30 kV. The step size for all analyses was 0.02°, and 2θ = 5° to 50°.

The relative crystallinity (*RC_FAU_*, %) referring to the FAU phase could be calculated as follows by the ASTM D3906-19 method, with adaptations [40]:

*%RC_FAU_* = *SFC* · *WF* · *(SX/SR)* · 100%, (1)

where *SFC* is the scale correction factor given by *SF_x_/SF_R_*, which is the ratio of the scale factor of the zeolite samples to the scale factor of the standard sample (ZX). The *SFC* was adopted as 1 because all samples were measured under the same conditions. The *WF* is the peak width factor obtained by the ratio of the FWHM (Full Width at Half Maximum) of the peak (*hkl* = 533) of the respective zeolite samples by the standard sample (ZX). And the *SX/SR* ratio is the sum of the heights of the eight most intense diffraction peaks (*hkl* = 331, 333, 440, 533, 642, 822, 555, 664) referring to each of the zeolite samples by the standard sample (ZX), respectively. These data were obtained from the measurements of background to maximum height for each of the eight peaks in each sample.

X-ray fluorescence (XRF) measurements were realized on a Bruker S2 Ranger (Atibaia, SP, Brazil), using an XFlash© Silicon Drift (Atibaia, SP, Brazil), Pd or Ag anode radiation, operating at 2 mA and 50 kV.

Scanning Electron Microscopy of high-resolution (FEG-SEM) images was obtained on a TESCAN MIRA 4 microscope (Brno, Tech Republic), using a secondary in-beam SE detector with an energy of 10 keV. The samples were coated with gold thin film deposition using a Denton Vacuum (Desk V model) evaporator(Brno, Tech Republic).

N_2_ adsorption-desorption isotherms were measured at −196 °C on a Micromeritics ASAP 2020 instrument (Norcross, GA, USA). Before the measurement, the samples were treated under a vacuum at 200 °C for 9 h to remove water and gases physisorbed.

The total pore volume (V_Total_) was determined at 0.99 of the relative pressure (p/p^0^). The specific surface area (S_BET_) was calculated using the Brunauer–Emmett–Teller (BET) method. The t-plot method was applied to calculate the external surface area (S_Ext._) and micropore volume (V_Micro._) [41]. The mesopore volume (V_Meso._) was calculated by the Barrett–Joyner–Halenda (BJH) model of desorption cumulative volume of pores [42]. N_2_ Tarazona NLDFT model (geometry of the pores: cylinder, type: DFT) was applied to calculate the pore size distributions [43,44].

The FTIR spectra were collected by Thermo Electron Corporation Spectrophotometer (Nicolet 6700 model, Waltham, MA, USA) in the mid-infrared region with a range between λ^−1^ = 400 to 4000 cm^−1^, accumulating 128 scans and 4 cm^−1^ of resolution. The samples were prepared with KBr and pressed into pellets.

Thermal Analyses were carried out using a TG/DTG apparatus (For zeolites: SDT/Q600 model, TA Instruments (New Castle, DE, USA; For residues: TG209F1 model, NETZSCH, Selb, Germany). The samples (5 mg) were heated in a flow of N_2_ (50 mL min^−1^) at a heating rate of 10 °C min^−1^ from room temperature to 800 °C.

## 3. Results and Discussion

### 3.1. Acid Leaching in Residue Glass Powder

XRF analyses of untreated (RGP) and acid-treated residue glass powder (TRGP01_25C and TRGP01_80C) are shown in Table 1. The results of the optimal temperature study indicate an increase in silicon percentage (%, wt.) and a decrease in sodium ions percentage (%, wt.) only with the effect of acid concentration. However, the temperature had the most significant influence on the removal of Ca^2+^, Mg^2+,^ and Fe^3+^ ions, resulting in a significant increase in the percentage of silicon.

Despite the partial leaching of Al^3+^ ions from the RGP in both acid treatments, the amount of aluminum required for synthesis was adjusted by the addition of sodium aluminate (the aluminum source). Therefore, only the minimum interference in the final amount of silicon and the maximum acid leaching of the Ca^2+^ cations from residue glass powder were evaluated.

In this way, the best experimental condition for the removal of Ca^2+^ cations from residue glass powder was studied by correlating treatment parameters such as time, concentration, and volume of HCl solution, fixing the temperature at 80 °C. XRF analyses of acid-treated residue glass powders (TRGP02) are shown in Table S1 (Supporting Information).

According to experimental values in Table 2, an enhancement from 43.06% to 56.60% of Ca^2+^ ions removal was observed due just to the variation of the acid concentration, fixing the time (60 min) and liquid/solid ratio (20 mL (2g)^−1^) for the samples TRGP02_1 and TRGP02_5, respectively. This percentage increase is even more considerable for varying HCl concentration, from 54.38% to 72.44% of removal, when evaluating samples TRGP02_2 and TRGP02_6, respectively, for a treatment time equal to 240 min.

The L/S ratio factor does not provide a significant effect on the percent removal, with a variation of -0.47% when increasing the ratio from 20 mL to 60 mL for the experiments with 3.0 mol L^−1^ and 240 min. On the other hand, greater treatment time and HCl concentration positively influence the removal of Ca^2+^ ions, with variations of +15.84% (for 3.0 mol L^−1^ and 20 mL) and +18.06% (240 min and 20 mL), respectively.

Thus, the experimental conditions that resulted in higher calcium removal were for sample TRGP02_6. This sample was chosen as the source of silicon in the syntheses of FAU zeolite (hereafter referred to as TRGP) because the acid leaching of calcium ions resulted in a higher silicon content compared to the untreated residue (RGP). In addition, the removal of Ca^2+^ ions is essential because in a system with other inorganic cations, such as Na^+^, the silicate species in solution can be modified by altering the crystallization process and reducing the yield of the desired phase, although the ΔH^∗^_f_ of Na-FAU is more favorable [45].

### 3.2. Synthesis of FAU-X Zeolite

The experiment results for Na-X seed crystals (ZX sample) are shown in the Supporting Information. Figure S1a presents the XRD result. The position and relative intensity of the peaks for the ZX seed sample match the hydrated Na-X zeolite from the IZA standard reference [2,46]. The characteristic peak at 2θ = 6.12° has d = 14.29 Å of the diffraction planes in [111] direction in cubic geometry. The Na-X crystals are composed of aggregated nanocrystals with an average diameter of 2 μm, as shown in the FEG-SEM image (Figure S1b). This spherical morphology is characteristic of the FAU-X zeolite. In addition, the elemental analysis by XRF (Table S2) of seed crystals indicates the formations of FAU-X framework with The Si/Al experimental ratio equal to 1.5 and a considerable percentage of Na^+^ ions as compensating cations in the zeolite structure. The supplemental analyses of the ZX sample were used to compare the material obtained in seed-assisted crystallization in the synthesis from residue RGP and TRGP.

#### 3.2.1. Effect of Seed Crystals Amount

The impurities in the residue make it difficult for the nucleation and growth steps of the crystals, so the hydrothermal syntheses of FAU-X were performed with untreated and acid-treated residues as an alternative source of silica. Different treatment times (12 h to 48 h) and seed loadings (0%, 1%, and 5% wt.) were analyzed to obtain pure-phase zeolites. Data from the Collection of Simulated XRD Powder Patterns For Zeolites [46] and the IZA Database of Zeolite Structures [2] were applied to identify the presence of crystalline phases in the as-synthesized materials. The three stronger peaks at a low-angle reflection that characterized the four zeolitic phases detected in this study are: 2θ = 6.12°, 10.00° and 11.73° for hydrated Na-X (FAU-X); 2θ = 12.46°, 21.67° and 28.10° for Na-P1 (GIS); 2θ = 7.20°, 10.19° and 12.49° for hydrated Linde Type A (LTA); 2θ = 14.16°, 24.65° and 31.99° for Sodalite Octahydrate (SOD) [46].

**Figure 1:**
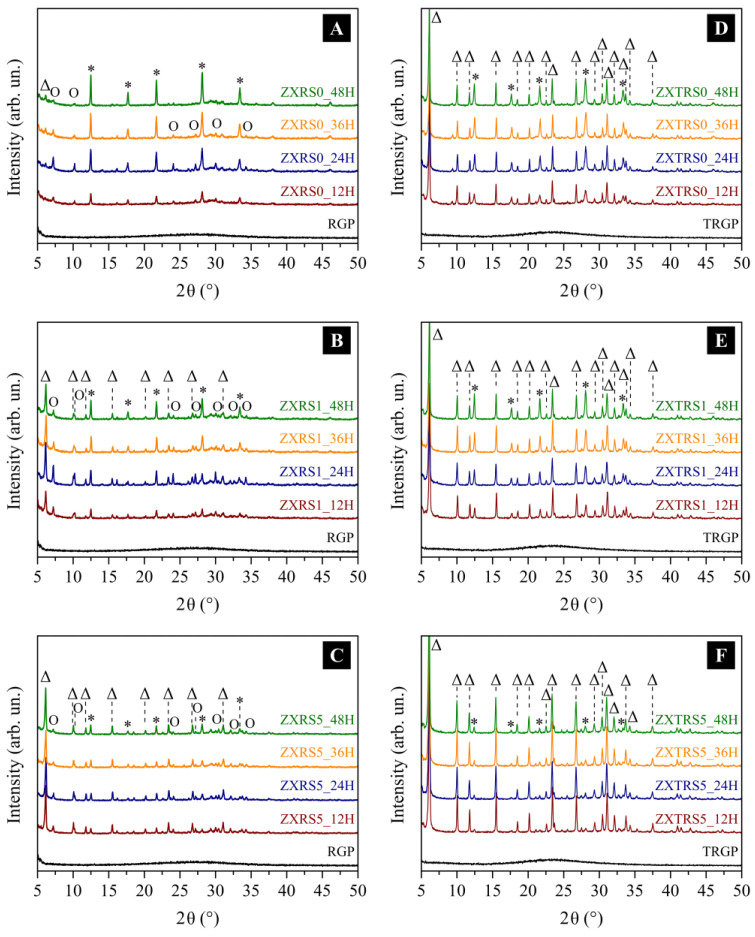
Powder XRD patterns of the zeolite samples from the untreated (RGP, ZXR zeolites) and acid-treated (TRGP, ZXTR zeolites) residues with different crystallization times (12 h, 24 h, 36 h, 48 h) and seed dosages: (**A**,**D**) S0 = 0% wt., (**B**,**E**) S1 = 1% wt., and (**C**,**F**) S5 = 5% wt. The symbols denote the following crystalline phases: (Δ) FAU, (∗) GIS, and (o) LTA.

Diffraction patterns relative to the synthesis of ZXRS0 from the untreated residue (RGP) are in Figure 1A. It is evident the presence of zeolite Na-P1 (GIS framework) as a primary phase, and their peaks intensify when the crystallization time increases (2θ = 12.45°, 17.69°, 21.63°, 28.12°, and 33.43°). Some peaks to the LTA framework as a second phase are more intense within 24 h but decrease in intensity by 48 h of crystallization. Only with 48 h of synthesis, the characteristic reflections of FAU framework are identified at 2θ = 6.14°, 15.48°, 23.39°, and 30.89°, and with low relative crystallinity (RC_FAU_ = 3%) (as seen in Table 3), beyond the GIS predominant phase. These results are consistent with zeolite production that does not use seeds to direct the growth of the desired structure and from RGP containing impurities and residual amorphous material.

In the case of synthesis from residues, the material composition and particle size are not homogeneous, making it difficult to produce pure phases in zeolites. Ostwald’s Step Rules cannot explain the coexistence of the GIS and LTA phases, since it would be expected to observe the appearance of the SOD and LTA phases, only after a certain synthesis time, with a decrease in the intensity of the diffraction signals related to the FAU phase. Thus, the presence of the GIS phase, mainly in the material synthesized without seeds in 12 h (ZXRS0_12H), can be related to the different dissolution ratios of the silicon source. According to the kinetic ternary phase diagrams of zeolite structures formulated by Maldonado et al. [47], single- and multi-phases can be correlated with the concentration of elements (Si, Al, NaOH molar fractions). Zeolites are classified in the GIS-SOD multi-phase region, in which these frameworks are the major phases, and a third phase or minor quantity of LTA (in Al-rich solution) and FAU (in Si-rich solution) have been identified.

However, in the ZXTRS0 synthesis from the acid-treated residue (TRGP) (Figure 1D), the zeolites contain FAU as the main and predominant phase at all synthesis times. According to diffraction patterns, the intensity of some eight peaks that define the FAU-X framework at 2θ = 15.5°, 26.7°, 30.4° and 33.7° increase along with the crystallization time, in which a maximum structural order is reached with RC_FAU_ equal to 46% at 48 h. Such a degree of crystallinity obtained for these samples was higher than the values obtained for the synthesis performed only with the glass waste without prior treatment (ZXRS0 synthesis), suggesting the efficiency of the acid treatment on the glass residue. Another secondary phase was identified related to Na-P1 (2θ = 12.45°, 17.69°, 21.63°, 28.12°, 33.43°), and without the presence of SOD and LTA frameworks.

New syntheses from non-treated and acid-treated residue glass powder were performed using crystal seed loadings to induce the growth of framework FAU. The low relative crystallinity values to materials from RGP with 1% wt. of seed (ZXRS1 samples, Table 3) denote the amount of residual amorphous material not transformed into the required structure and a poor degree of structural arrangement for the faujasite phase. In XRD analysis (Figure 1B), the greater RC_FAU_ percentage was only 24 h of synthesis in which a phase competition between Na-X and Na-P1 (GIS, 2θ = 12.50°, 17.69°, 21.70°, 28.12°, 33.43°) is perceived, and the presence of zeolites A (LTA, 2θ = 7.24° with d_200_ = 12.21 Å) and sodalite (SOD, 2θ = 14.09°) as a third and fourth phase. Despite the decrease of RC_FAU_ from 15% to 12%, only after 48 h of synthesis does the intensity of the peaks related to LTA decrease, revealing a greater presence of the FAU and GIS structures. Such fact is mainly indicated by the characteristic reflections for the FAU zeolite around 2θ = 6.18°, 10.07° and 11.80° (d_111_ = 14.31 Å, d_22_0 = 8.79 Å, d_311_ = 7.50 Å, respectively), and for the Na-P1 zeolite around 2θ = 12.50°, 21.70° and 28.12° (d = 7.08 Å, 4.09 Å and 3.17 Å).

However, the XRD patterns to zeolite from TRGP with 1% wt. of seed (ZXTRS1 samples) reveal that at all crystallization times showed the faujasite phase as the main and predominant one, with the diffractogram profile similar to that of the NaX zeolite standard sample, and a secondary phase relative to Na-P1 zeolite without the presence of LTA and SOD structures (Figure 1E). Furthermore, the efficiency of the acid treatment of the residue to reduce the impurities present in the material made such an effect possible, even though the same percentage of seeds (on a dry basis) was used for both syntheses (ZXRS1 and ZXTRS1). The percentage decrease of the RC_FAU_ for these synthesized samples indicates that with a short crystallization time, it is possible to obtain the desired structure with a high degree of structural order (12 h with RCFAU = 52%). It should be noted that longer times lead to the formation of the GIS structure, which is the most stable phase.

As shown in Figure 1C,F, the syntheses were carried out using 5% of crystal seeds from untreated (ZXRS5) and acid-treated (ZXTRS5) RGP. The aim was to obtain single-phase and high-crystallinity zeolites. The RC_FAU_ values around 77–81% for the ZXTRS5 samples suggest the efficiency of acid leaching on residue in which 12 h of synthesis obtained a high-ordering FAU zeolite. The longer times lead to the decrease of RC_FAU_ values for both syntheses (20% to 17% for ZXRS5 synthesis and 81% to 77% for ZXTRS5 synthesis). Also, the intensification of the peaks for Na-P1 zeolite (2θ = 12.4°, 17.7°, 21.6°, 28.0°, 33.3°) in the time range studied means the transformation of one part of the Na-X zeolite into the GIS structure which is the most stable and dense phase. The GIS framework remains to a lesser extent due to the presence of the seed. According to Cundy and Cox [38], the seed eliminated primary nucleation and reduced induction time by providing adequate surface area for crystal growth. In our study, when comparing the ZXTRS5_12H sample with others, the use of seeds allowed the crystal growth to be directed to the FAU phase, thus reducing the crystallization time to the Na-X zeolite formation.

Then, the seed-assisted technique and the use of alternative sources of silicon have great potential for the development of more economical production routes since these environmental liabilities will be transformed into commercially valuable materials [9,48].

#### 3.2.2. Influence of Cations in Framework Type

Acid leaching of the residual glass powder with appropriate parameters promoted a higher percentage of Na^+^ ions than Ca^2+^ ions. Thus, according to the elemental composition by XRF analysis for the synthesized samples (S.I. in Table S2), these results were confirmed by the percentage of cations in each sample and the similar experimental Si/Al ratios for the same synthesis group (ZXRS0, ZXRS1, and ZXRS5 samples; ZXTRS0, ZXTRS1, and ZXTRS5 samples). Also, lower experimental Si/Al ratio values when using acid-treated residue indicate a lower presence of amorphous material not transformed into zeolite that can affect this conversion ratio and a better dissolution of acid-treated residue as a gel form that has been transformed into a crystalline structure.

The decisive formation of Na-X zeolite (FAU) was caused by the presence of Na^+^ ions as a mineralizing agent, making it the compensating cation of the FAU framework for all synthesis from TRGP. Also, the high content of Ca^2+^ cations in the synthesis from RGP interceded on the targeting of the as-synthesized zeolite to the predominant GIS framework.

The recommended range of mineralizing agent (OH^−^ ions) for the ideal nucleation rate of Na-X zeolite by standard synthesis is between 2–3 mol NaOH L^−1^. If the found value is above, the zeolite analcime (ANA) or sodalite (SOD) is obtained, and if it is below, the FAU nucleation rate goes to zero, obtaining the GIS [39]. However, the values for these syntheses are within this range. It is very likely that the considerable percentage of calcium ions on the residual glass powder has induced the formation of Na-P1 zeolite (GIS), mainly on the untreated material. Since this inorganic cation is a driver to the more stable phase, the GIS phase grows faster than the Na-X zeolite (FAU) [39].

According to Pace et al. [49], the nature of the compensating cation explains the influence on the intra-reticular ratio of SiO_2_/Al_2_O_3_. So, the monovalent cations lead to the formation of zeolites richer in silicon than bivalent cations to compensate for the negative charges of the structure. The presence of Ca^2+^ ions due to the chemical composition of the residue glass powder interfered with obtaining a single-phase material.

#### 3.2.3. Interzeolite Transformations

Some critical factors could be considered in the evaluation for obtaining the required product: molar composition of the synthesis gel, crystallization time, amount of mineralizing agent, and the possible phase competition between FAU and the LTA, SOD, GIS, and ANA structures [39].

The molar proportion of gel syntheses for all sample studies is within the range adopted by the standard methodology to obtain Na-X zeolite easily. Regarding the crystallization time, obtaining FAU zeolites with 12 h from treated waste glass proved to be effective considering the decrease of the crystallization time of the adapted methodology from 24 h to 12 h [39] and the considerable values of RC_FAU_, mainly due to the increase of the content of seed from 0% wt. to 5% wt. On the other hand, a longer crystallization time is necessary to obtain the FAU zeolite from the untreated residue, requiring the addition of a larger concentration of seed to decrease this time.

**Figure 2:**
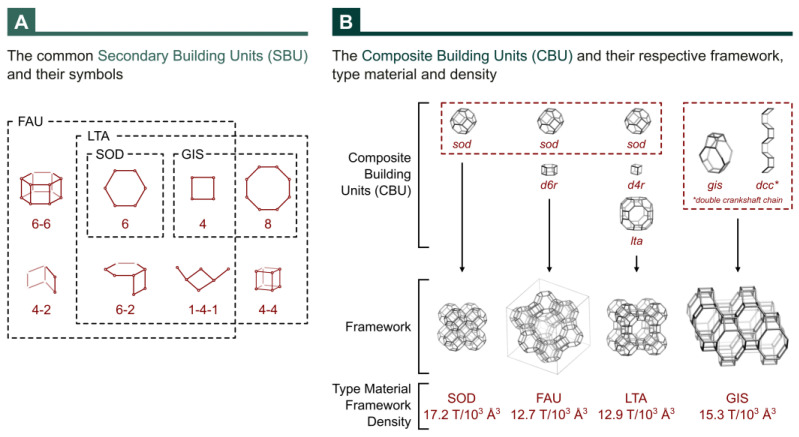
Scheme of common secondary building units (**A**) and Composite Building (**B**) for Faujasite (FAU), Linde Type A (LTA), Gismondine (GIS), and Sodalite (SOD) framework types. Framework and CBU images adapted from Atlas of Zeolite Framework Types [1].

As shown in Figure 2A, all the identified phases have secondary building units in common. Furthermore, there are a few options for connecting the sodalite cavity (β-cavity) (Figure 2B): directly through its square face generating the mineral sodalite or through the square face, with a square prism generating zeolite A and through the hexagonal face with a hexagonal prism generating the zeolite faujasite (X or Y, depending on the Si/Al ratio present in the material) [49]. Therefore, the formation of a material with more than one phase is very likely to occur due to the impurity of the glass powder, which interferes with the nucleation and growth of crystals. However, no change in the molar composition of the reaction mixture occurs.

Ostwald’s Step Rules state that successive intermediate crystalline phases are produced until the thermodynamically stable phase is formed [38]. These phase transitions are consistent with the enthalpy of formation (ΔH_f_ − ΔH°) relative to the phase formed and occur with increasing synthesis temperature and/or time, starting from low to high framework density (e.g., LTA → FAU → GIS → ANA) [47].

Thus, for the crystallization interval studied from 12 h to 48 h, the transformation of one part of the structure into another occurred with increasing time, with the intensification of the characteristic peaks of the GIS structure for all syntheses with both residual silicon sources. However, the addition of seed (from 0% to 5%) caused a lower presence of the GIS structure, especially for the synthesis in which the residue glass powder presents fewer impurities.

According to Figure 2B, the Na-P1 zeolite presents the GIS framework with a framework density of around 15.3 T-atoms per 1000 Å^3^, a higher value than for the Na-X zeolite (FAU), with 12.7 T-atoms per 1000 Å^3^. Hence, the extended crystallization time made possible the rearrangement of the silicon-aluminum tetrahedron (TO_4_) in which the secondary building units become the units that form the GIS structure (4 or 8) instead of being the unit’s characteristic of faujasite (6-6 or 6-2 or 6-2 or 4-2 or 1-4-1 or 4), as shown in Figure 2A.

Sayehi et al. [16] obtained similar results with a mixture of FAU and GIS phases in the synthesis of zeolite from two types of waste, aluminum scrap and fluorescent tubes, but without applying the seeding technique. According to these authors, prolonged synthesis times favored the transformation of the metastable phase of the Na-FAU zeolite to the more stable and denser phase of Na-P1 zeolite, with the new phase formed by dissolving the previous phase in a supersaturated solution [16].

### 3.3. Characterization of Samples ZXRS5 and ZXTRS5

**Figure 3:**
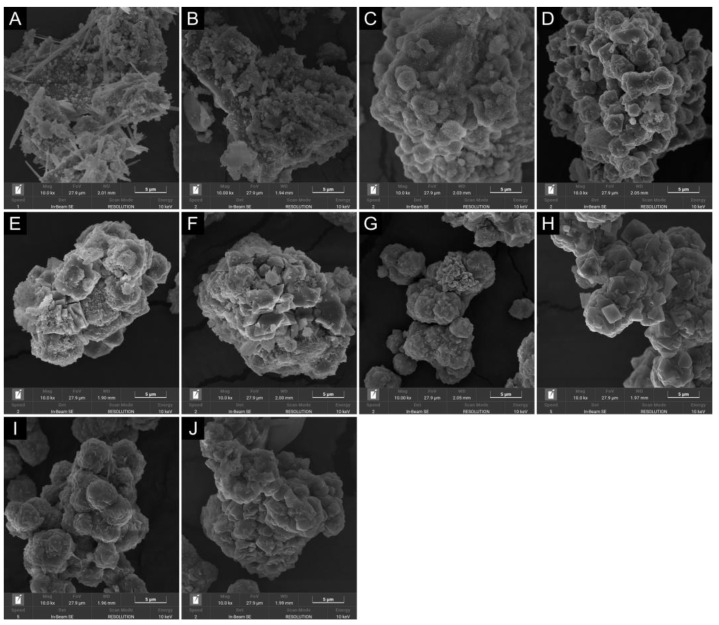
FEG-SEM images of (**A**) untreated and (**B**) acid-treated residues; ZXRS5 zeolite samples: (**C**) 12 h, (**D**) 24 h, (**E**) 36 h, and (**F**) 48 h; and ZXTRS5 zeolite samples: (**G**) 12 h, (**H**) 24 h, (**I**) 36 h, and (**J**) 48 h.

To understand the crystallization process at different stages by adding 5% wt. of seed, the morphology of the zeolites was followed by scanning electron microscopy of high resolution (FEG-SEM). Figure 3A shows the image of the untreated residual glass powder. The morphology resembles a mixture of various sizes of disordered thin nanorods and spherical aggregates, with such behaviors typical of amorphous material. The acid-leaching procedure results in small particles as worm-like form clusters (Figure 3B). It is possible that these particles may have dissolved more easily than the large ones present in the RGP.

According to the previous results [16,17,50,51], the Na-A, Na-X, and Na-P1 zeolites could have the following morphologies: cubic, octahedral, and spherical particles, respectively. Thus, after 12 h of synthesis from RGP, the crystallization process results in small condensed spheres with a sponge-like surface appearance (Figure 3C). XRD data indicate a mixture of FAU, LTA, and GIS crystalline phases (Figure 1C) and an RC_FAU_ percentage of 20%. Otherwise, changing the amorphous precursor for TRGP achieves an almost pure FAU phase with 81% RC_FAU_, as well as morphology and microporous structure similar to Na-X seed crystals (Figure 3G).

Long crystallization times lead to changes in the morphology of the samples, indicating the transformation of metastable phases with similar SBU and CBU. The SEM images for zeolites with low RC_FAU_ percentage reveal condensed polycrystalline aggregates with different shapes (Figure 3D–F, 24 h to 48 h), which is consistent with a mixture of GIS, LTA, and FAU phases detected by XRD analysis (Figure 1C). However, for the sample with high FAU phase content (ZXTRS5), the decrease in RC_FAU_ occurred, and the morphology changed slightly. The increase in the presence of the GIS phase for longer synthesis times detected by XRD analysis (Figure 1F) is corroborated by SEM images. In this case, small spherical aggregate particles with irregular sizes were observed (Figure 3H–J, 24 h to 48 h). It is also observed that the zeolitic crystal aggregates are similar in size to the residual agglomerates. This fact reinforces the concept that the TRGP/RGP particles do not dissolve completely and that the crystallization process of the crystals takes place simultaneously under these particles.

**Figure 4:**
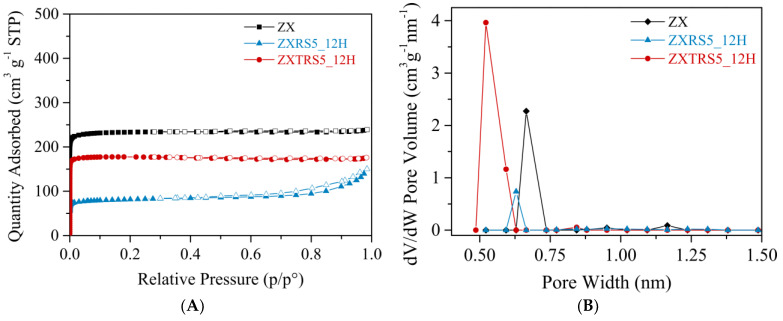
(**A**) N_2_ adsorption-desorption isotherms, and (**B**) the pore size distribution of zeolites samples: standard zeolite sample (ZX); zeolite samples from untreated and acid-treated residues with 5% wt. of seed crystals (ZXRS5_12H and ZXTRS5_12H, respectively). The solid symbols denote the adsorption data, while the hollow symbols denote the desorption data.

Other characterization techniques were used to analyze the best sample from the acid-treated residue (ZXTRS5_12H zeolite) and two other selected samples from the untreated residue (ZXRS5_12H zeolite) and the crystal seeds (ZX zeolite). The N_2_ adsorption-desorption isotherms and their respective curves of pore size distributions are reported in Figure 4. The data on textural properties are summarized in Table 4.

The presence of microporous in ZX and ZXTRS5_12H samples is indicated by the reversible type I(a) isotherms. These isotherms are given by microporous materials having mainly narrow micropores (<~1 nm) [52]. For zeolite from the acid-treated residue, the measured S_BET_, S_Ext._, V_Micro_, and pore size are close to the values obtained for ZX material. These results are consistent for FAU zeolites, especially when compared to the data for the commercial FAU sample and the as-synthesized zeolites from aluminoborosilicate glass prepared by Tsujiguchi and co-authors [31].

The lower presence of Na-X zeolite caused the appearance of hysteresis in the isotherm curve for the ZXRS5_12H sample, and consequently, the higher presence of Na-P1 zeolite and a residual amorphous material that was not converted into zeolite. The hysteresis loop is type H4, often found in aggregated crystals and some mesoporous zeolites. This loop corresponds to the adsorption branch composed of Type I and II, with more pronounced adsorption at low p/p^0^ associated with the filling of micropores and at high relative pressures, indicating that the sample contains mesopores [52]. The different morphologies observed in the sample ZXRS5_12H compared to the ZXTRS5_12H sample (Figure 3) corroborate the introduction of a certain amount of mesopores, as evidenced by the increased external surface area and mesopore volume in Table 4.

**Figure 5:**
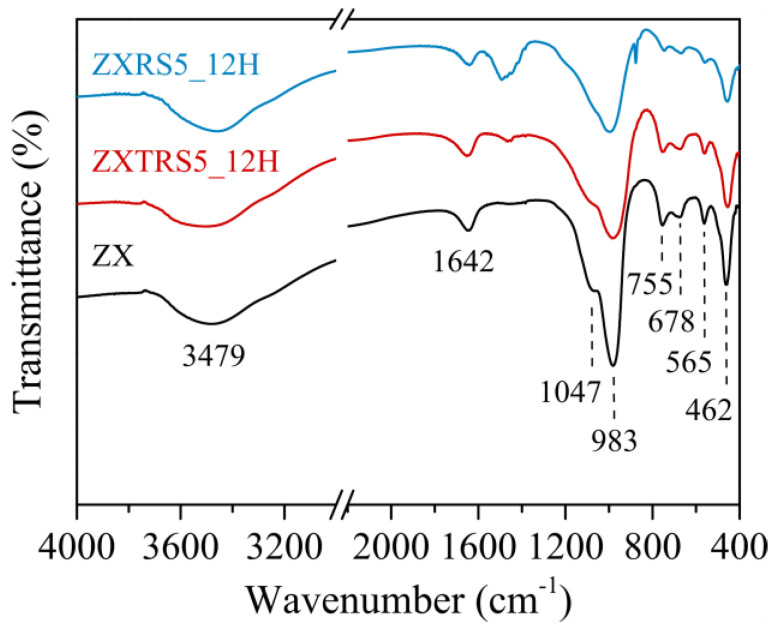
FTIR spectra of zeolite samples: standard zeolite sample (ZX); zeolite samples from untreated (ZXRS5_12H) and acid-treated (ZXTRS5_12H) residues with 5% wt. of seed crystals in 12 h of synthesis.

The FTIR spectra of the synthesized zeolites (ZX, ZXTRS5_12H, and ZXRS5_12H samples) are exhibited in Figure 5. The bands at 3479 cm^−1^ and 1642 cm^−1^ are characteristics of stretching (ν O-H) and bending vibrations (δ H-O-H) of H_2_O molecules adsorbed in cavities and channels of zeolites. The absorption range between 1300 cm^−1^ to 400 cm^−1^ contains the external and internal vibrations characteristics of tetrahedral TO_4_ (with T-atoms = Si or Al) in zeolites [16,17,51,53]. For ZX zeolite, the O-T-O bonds in tetrahedral are marked by the absorption bands associated with the asymmetric (ν_asy_) and symmetric (ν_sy_) intratetrahedral stretching vibrational modes at 983 cm^−1^ and 678 cm^−1^, respectively. The band at 462 cm^−1^ is relative to the bending vibration modes of O-T-O bonds. The intertetrahedral vibrations are located in the spectrum at 1047 cm^−1^ (ν_asy_) and 755 cm^−1^ (ν_sy_). The region around 650 cm^−1^ to 500 cm^−1^ is related to the bands originating from structural units in zeolites [53]. Thus, the medium peak at 565 cm^−1^ is assigned to double 6-membered rings (D6R) characteristic of faujasite.

The less intense and broader peaks visualized for the zeolite ZXTRS5_12H correspond to those defined for ZX. This sample contains RC_FAU_ = 81% and a small amount of GIS phase. The appearance of the weak and broad peak at ca. 1462 cm^−1^ can be attributed to carbonate species derived from the silicon source (TRGP) [16,51]. The presence of the other phases (Na-A and Na-P1 zeolites), the impurities from the untreated residual glass powder and a minor RC_FAU_ (20%) caused a change in the spectrum for the zeolite ZXRS5_12H: two weaker shoulders between 1210 cm^−1^ and 1084 cm^−1^ can be characterized as zeolite Na-A; the medium and broader peak at ca. 1462 cm^−1^ for carbonate species; and the decrease of the bands in a range between 800 cm^−1^ to 400 cm^−1^.

The thermal complementary analysis is attached to the supporting information (SI). Similar thermogravimetric curves are observed for ZX and ZXTRS5_12H zeolites (Figure S2A), with a loss of approximately 25–27% of the mass in two stages. The samples lost H_2_O molecules physisorbed on the channels and cavities between 20 °C and 100 °C. In the second step, the H_2_O molecules strongly adsorbed were eliminated at 100 °C to 200 °C. The ZXRS5_12H zeolite loses 24% of its mass in three stages. The first and second steps are the same as already defined for the other two samples. On the other hand, the third step between 400 °C and 600 °C can be associated with the impurities and residual amorphous material that has not been converted into zeolite (curve for RGP02_6 sample in Figure S2B).

## 4. Conclusions

The eco-friendly syntheses are more sustainable and economical synthetic routes that use alternative sources of precursors. In this study, we obtained zeolite with higher crystallinity for the FAU phase using seed-assisted hydrothermal synthesis from converting residual colorless glass powder. The best result for the as-synthesized zeolite was obtained from the acid-treated residue, adding 5% wt. of seeds and 12 h of crystallization.

The study revealed that the presence of different ions as compensating cations influenced the final framework type. Acid leaching promoted a higher percentage of Na^+^ ions than Ca^2+^ ions in the residue glass powder. Consequently, the monovalent cation was the mineralizing agent for the FAU phase, and the bivalent cation interceded in the targeting of the GIS phase in the syntheses from acid-treated and untreated residues, respectively. Moreover, interzeolite transformations occurred with increasing synthesis time. Also, the complementary analyses reveal the morphological and textural properties of the synthesized zeolites, in which the structure obtained with a high RC_FAU_ value is similar to Na-X seed crystals. Therefore, the seeding technique combined with acid leaching was fundamental in targeting the FAU phase in the synthesis of waste material.

## Figures and Tables

**Table 1:** XRF analysis of residue glass powder before (RGP) and after the acid leaching at 25 °C (TRGP01_25C) and 80 °C (TRGP01_80C).

Samples	Chemical Composition (%, wt.)							
	SiO_2_	Al_2_O_3_	Na_2_O	CaO	MgO	Fe_2_O_3_	K_2_O	Others
RGP	66.94	3.31	8.80	14.84	3.60	0.67	0.67	1.17
TRGP01_25C	78.76	3.22	2.50	10.11	3.10	0.49	0.37	1.45
TRGP01_80C	87.50	2.50	2.50	4.29	2.00	0.35	0.19	0.67

**Table 2:** Experimental values for acid leaching procedure (concentration of HCl mol/L, liquid/solid ratios mL (2 g)^−1^, and treatment time min) and responses in terms of calcium ions removed (%).

Samples	[HCl] (mol L^−1^)	L/S ratios ^a^ (mL (2g)^−1^)	Time (min)	Ca^2+^ Removed (%)
TRGP02_1	0.50	20	60	43.06
TRGP02_2	0.50	20	240	54.38
TRGP02_3	0.50	60	60	55.26
TRGP02_4	0.50	60	240	69.27
TRGP02_5	3.00	20	60	56.60
TRGP02_6	3.00	20	240	72.44
TRGP02_7	3.00	60	60	56.33
TRGP02_8	3.00	60	240	71.97

^a^ L/S ratio: liquid/solid ratios, with the liquid being the volume of HCl solution (mL) and the solid being the mass of residue glass powder (value fixed in 2 g).

**Table 3:** Summary of relative crystallinity values to FAU phase (RC_FAU_, %) of the samples synthesized from the untreated (RGP) and acid-treated (TRGP) residues with different seed dosages (0% wt., 1% wt., and 5% wt.) and crystallization times (12 h, 24 h, 36 h, and 48 h).

Time	Relative Crystallinity to FAU Phase (RC_FAU_, %)					
	0% wt.		1% wt.		5% wt.	
	RGP	TRGP	RGP	TRGP	RGP	TRGP
12 h	0	39	8	52	20	81
24 h	0	39	15	49	18	77
36 h	0	40	12	51	15	78
48 h	3	46	12	42	17	77

**Table 4:** Textural properties of zeolite samples: standard zeolite sample (ZX); zeolite samples from untreated and acid-treated residues with 5% wt. of seed crystals in 12 h of synthesis (ZXRS5_12H and ZXTRS5_12H, respectively).

Samples	S_BET_^a^ (m^2^ g^−1^)	S_Ext._^b^ (m^2^ g^−1^)	V_micro_^b^ (cm^3^ g^−1^)	V_meso_^b,c^ (cm^3^ g^−1^)	V_meso_^d^ (cm^3^ g^−1^)	Pore Size ^e^ (nm)
ZX	967	61	0.335	0.035	-	0.67
ZXTRS5_12H	742	45	0.258	0.014	-	0.52
ZXRS5_12H.	323	53	0.103	0.129	0.122	0.63

Data was collected using the following methods: ^a^ BET method and ^b^ t-plot method. ^c^ V_meso_ = V_total_ − V_micro_. ^d^ BJH desorption cumulative volume of the pore. ^e^ N_2_ Tarazona NLDFT model. - Not applicable.

## Data Availability

The original contributions presented in this study are included in the article/Supplementary Materials. Further inquiries can be directed to the corresponding author.

## Supplementary Materials

The following supporting information (SI) can be downloaded at https://www.mdpi.com/article/10.3390/ma18071393/s1, Table S1: XRF analysis of acid-treated residue glass powder (TRGP); Figure S1: (A) Powder XRD patterns, and (B) FEG-SEM image of the zeolite Na-X seed (ZX); Figure S2: Thermogravimetric analysis (TA) and derivative (DTG) curves: (A) standard zeolite sample (ZX); zeolite samples from untreated (ZXRS5_12H) and acid-treated (ZXTRS5_12H) residues with 5% wt. of seed in 12 h of synthesis; (B) untreated (RGP) and acid-treated (TRGP02_6) residue glass powder; Table S2: XRF analysis of zeolites samples (Silicon source: silica (ZX) or untreated (ZXR) and acid-treated (ZXTR) residue glass powder).
