# Supplementary material for: Seed-Assisted Crystallization in the Hydrothermal Synthesis of FAU Zeolite from Acid-Treated Residue Glass Powder
Source: Materials (Basel). 2025 Mar 21;18(7):1393. doi: 10.3390/ma18071393 (PMC11989644; doi:10.3390/ma18071393)
Supplement: Supplementary file 1 [file materials-18-01393-s001.zip › materials-3490780-supplementary.pdf]

Supporting Information

Seed-Assisted Crystallization in the Hydrothermal Synthesis of FAU Zeolite from Acid-Treated Residue Glass Powder

Paulla B. F. Sousa [1,2], Lindiane Bieseki [1], and Sibele B. C. Pergher [1,*]

[1] Laboratory of Molecular Sieves (LABPEMOL), Institute of Chemistry, Federal University of Rio Grande do Norte, Natal 59078-970, RN, Brazil
[2] Laboratory of Catalysis, Instituto Nacional de Tecnologia (INT), Rio de Janeiro 20081-312, RJ, Brazil
* Correspondence: sibelepergher@gmail.com

1. Experimental data: additional results

Additional experimental details for residue glass powders and as-synthesized zeolite materials.

ACID LEACHING ON RESIDUE GLASS POWDER

Experimental data of the study of the optimal experimental condition for acid leaching in residue glass powder: elemental composition by XRF analysis in Table S1.

Table S1: XRF analysis of acid-treated residue glass powder (TRGP).

Samples	Chemical composition (%, wt.)							
	SiO_2	Al_2O_3	Na_2O	CaO	MgO	Fe_2O_3	K_2O	Others
TRGP02_1	83.37	2.41	1.90	8.45	2.00	0.63	0.25	0.99
TRGP02_2	84.91	2.52	2.00	6.77	1.80	0.63	0.23	1.14
TRGP02_3	84.07	2.51	2.50	6.64	2.30	0.52	0.19	1.27
TRGP02_4	87.14	2.64	2.10	4.56	2.00	0.47	0.17	0.92
TRGP02_5	85.31	2.39	2.00	6.44	2.00	0.46	0.21	1.19
TRGP02_6	88.63	2.48	1.70	4.09	1.70	0.33	0.17	0.90
TRGP02_7	85.19	2.48	2.00	6.48	2.00	0.45	0.21	1.19
TRGP02_8	88.16	2.45	2.20	4.16	1.80	0.33	0.16	0.74

HYDROTHERMAL SYNTHESIS OF NA-X CRYSTAL SEEDS

Experimental data for Na-X zeolite (sample ZX) employed in hydrothermal synthesis as seed crystals: powder XRD patterns in Figure S1A and scanning electron microscopy of high resolution in Figure S1B.

Figure S1: (**A**) Powder XRD patterns and (**B**) FEG-SEM image of the zeolite Na-X seed (ZX).

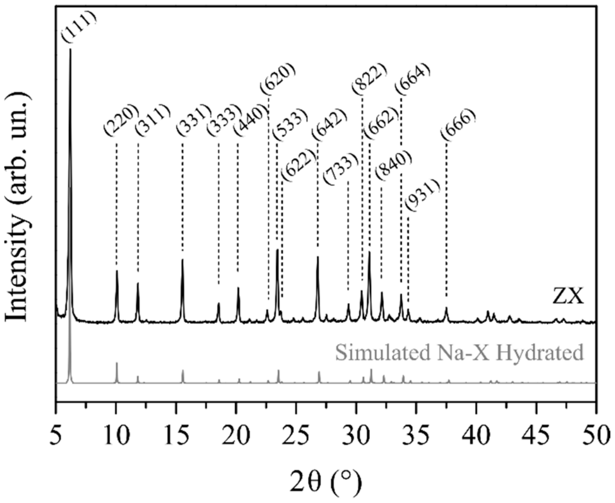

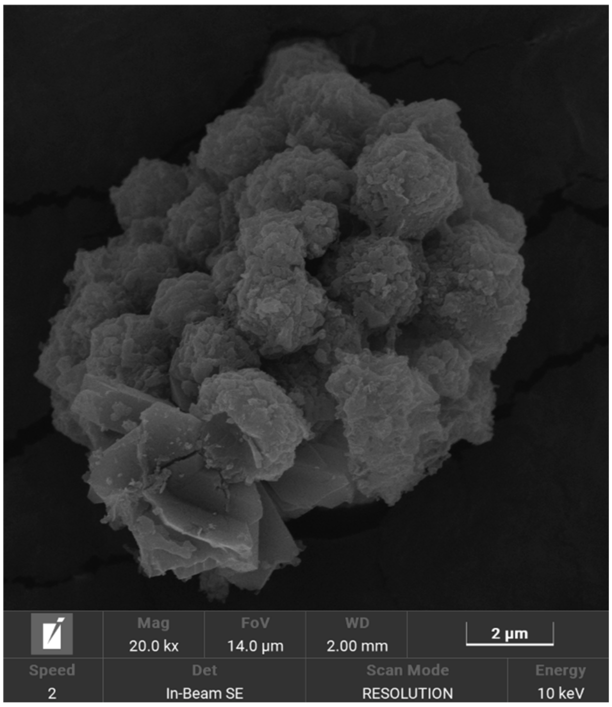

(A) **(B)**

SYNTHESIS OF FAU ZEOLITE FROM UNTREATED AND ACID-TREATED RESIDUES AND DIFFERENT SEED PERCENTAGES

Experimental data for zeolites from untreated (samples ZXR) and acid-treated (samples ZXTR) residue glass powders: thermal behavior accompanied by thermogravimetric and derivate curves for selected samples in Figure S2 and elemental composition by XRF analysis in Table S2.

Figure S2: Thermogravimetric analysis (TA) and derivative (DTG) curves: (**A**) standard zeolite sample (ZX); zeolite samples from untreated (ZXRS5_12H) and acid-treated (ZXTRS5_12H) residues with 5% wt. of seed in 12 h of synthesis; and (**B**) untreated (RGP) and acid-treated (TRGP02_6) residue glass powders.

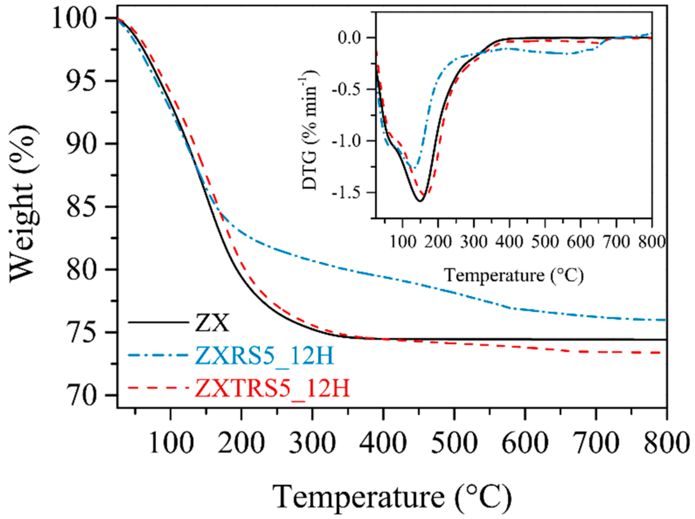

(A)

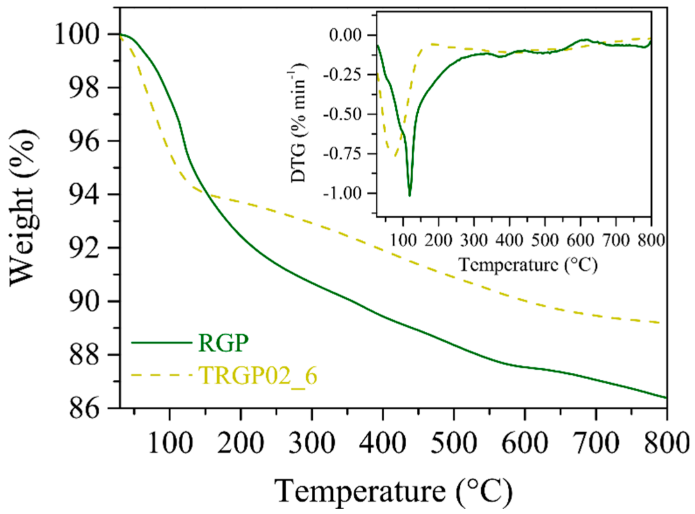

(B)

Table S2: XRF analysis of zeolite samples (Silicon source: silica (ZX) or untreated (ZXR) and acid-treated (ZXTR) residue glass powders).

Samples	Si/Al ratios	Chemical composition (%, wt.)							
		SiO_2	Al_2O_3	Na_2O	CaO	MgO	Fe_2O_3	K_2O	Others
ZX	1.5	53.05	30.81	12.90	-	2.70	0.02	0.11	0.41
ZXRS0_12H	3.2	51.65	13.90	9.50	19.25	3.50	0.83	0.42	0.95
ZXRS0_24H	2.3	48.62	18.22	11.10	16.56	3.50	0.73	0.45	0.82
ZXRS0_36H	2.7	50.27	15.57	9.20	19.58	3.50	0.88	0.32	0.68
ZXRS0_48H	2.8	51.35	15.27	9.90	18.74	3.00	0.87	0.35	0.52
ZXRS1_12H	3.1	51.73	14.20	6.90	21.76	3.70	0.97	0.29	0.45
ZXRS1_24H	2.1	49.64	20.16	10.40	15.27	3.20	0.66	0.24	0.43
ZXRS1_36H	2.6	50.53	16.56	8.60	19.25	3.40	0.86	0.28	0.52
ZXRS1_48H	2.4	49.80	17.61	10.10	17.81	3.20	0.80	0.27	0.41
ZXRS5_12H	2.7	54.11	17.03	6.00	18.64	2.70	0.82	0.25	0.45
ZXRS5_24H	2.5	53.39	18.12	6.70	17.64	2.50	0.83	0.24	0.58
ZXRS5_36H	2.8	54.01	16.29	5.90	19.81	2.50	0.96	0.25	0.28
ZXRS5_48H	2.5	53.06	17.78	6.40	18.44	2.70	0.87	0.28	0.47
ZXTRS0_12H	1.6	52.39	27.08	9.00	7.99	2.40	0.52	0.13	0.49
ZXTRS0_24H	1.6	51.23	27.32	9.90	7.81	2.80	0.36	0.18	0.40
ZXTRS0_36H	1.6	51.69	27.51	10.20	7.70	2.00	0.34	0.17	0.39
ZXTRS0_48H	1.5	47.71	27.79	11.50	7.15	4.80	0.31	0.30	0.44
ZXTRS1_12H	1.8	52.92	25.31	9.40	8.51	2.60	0.53	0.23	0.50
ZXTRS1_24H	1.8	51.47	25.00	11.00	7.88	3.60	0.49	0.20	0.36
ZXTRS1_36H	1.7	53.10	26.85	9.40	7.98	1.80	0.34	0.12	0.41
ZXTRS1_48H	1.7	52.69	26.68	9.90	7.86	1.80	0.35	0.16	0.56
ZXTRS5_12H	1.6	53.03	27.76	9.80	6.56	1.90	0.31	0.19	0.45
ZXTRS5_24H	1.6	51.33	27.46	10.60	6.51	3.10	0.33	0.27	0.40
ZXTRS5_36H	1.5	50.35	28.47	11.00	5.69	3.40	0.24	0.30	0.55
ZXTRS5_48H	1.8	56.06	27.04	7.00	7.48	1.60	0.39	0.10	0.33

- : Ion species not found.
